# Towards Low-Cost Yet High-Performance Sensor Networks by Deploying a Few Ultra-fast Charging Battery Powered Sensors

**DOI:** 10.3390/s18092771

**Published:** 2018-08-23

**Authors:** Qing Guo, Wenzheng Xu, Tang Liu, Hongyou Li, Zheng Li, Jian Peng

**Affiliations:** 1 College of Computer Science, Sichuan University, Chengdu 610065, China; qingguo45187@163.com (Q.G.); wenzheng.xu3@gmail.com (W.X.); lihongyou@scu.edu.cn (H.L.); lizheng@scu.edu.cn (Z.L.); 2 College of Fundamental Education, Sichuan Normal University, Chengdu 610068, China; liutang80@hotmail.com

**Keywords:** rechargeable sensor networks, heterogeneous sensor network, ultra-fast sensors, off-the-shelf sensors, joint charging scheduling, routing allocation algorithm

## Abstract

The employment of mobile vehicles to charge sensors via wireless energy transfer is a promising technology to maintain the perpetual operation of wireless sensor networks (WSNs). Most existing studies assumed that sensors are powered with off-the-shelf batteries, e.g., Lithium batteries, which are cheap, but it takes some non-trivial time to fully charge such a battery (e.g., 30–80 min). The long charging time may incur long sensor dead durations, especially when there are many lifetime-critical sensors to be charged. On the other hand, other studies assumed that every sensor is powered with an ultra-fast charging battery, where it only takes some trivial time to replenish such a battery, e.g., 1 min, but the adoption of many ultra-fast sensors will bring about high purchasing cost. In this paper, we propose a novel heterogeneous sensor network model, in which there are only a few ultra-fast sensors and many low-cost off-the-shelf sensors. The deployment cost of the network in the model is low, as the number of ultra-fast sensors is limited. We also have an important observation that we can significantly shorten sensor dead durations by enabling the ultra-fast sensors to relay more data for lifetime-critical off-the-shelf sensors. We then propose a joint charging scheduling and routing allocation algorithm, such that the longest sensor dead duration is minimized. We finally evaluate the performance of the proposed algorithm through extensive simulation experiments. Experimental results show that the proposed algorithm is very promising and the longest sensor dead duration by it is only about 10% of those by existing algorithms.

## 1. Introduction

Wireless sensor networks are widely used in many Internet of Things (IoTs) applications, including video monitoring, traffic control, structural health monitoring, radiation detection, forest fire and volcanor monitoing, etc. [1,2,3,4,5]. The energy consumption of sensors on data transmission however is very high. Although many techniques have been proposed to save sensor energy, such as dynamic duty cycle [6,7,8], sensors will run out of their energy eventually.

Many researchers proposed to prolong sensor lifetimes by enabling them to harvest energy from their surrounding environments, such as solar energy, wind energy, etc. [9,10]. However, the energy harvesting rates of sensors are low and unstable, due to their dynamic surrounding environments. For example, it is reported that the energy generating rates in sunny, cloudy and shadowy days can vary by up to three orders of magnitude in a solar harvesting system [11]. Such unpredictability and intermittency pose challenges in the efficient usage of harvested energy for various monitoring or surveillance tasks [12]. Recently, some pioneering researchers [13,14,15,16] proposed a revolutionary way to replenish sensor energy, that is, employing a mobile vehicle equipped with a charging device to move in the vicinity of a lifetime-critical sensor, and charge it via wireless energy transfer [14]. By doing so, the charging rate is high and stable.

A lot of attention has been paid to the vehicle charging scheduling for wireless sensor networks. Most of these studies assumed that sensors are powered with off-the-shelf batteries [15,17,18,19,20,21,22,23,24,25], such as Lithium batteries, we here abbreviate an off-the-shelf battery powered sensors by an off-the-shelf sensor. The price of such a battery is only about a few dollars [26]; however, it usually takes some non-trivial time to fully charge one, e.g., 30–80 min [26]. Therefore, these studies mainly focused on shortening sensor dead durations due to the long charging time of off-the-shelf sensors.

**Figure 1:**
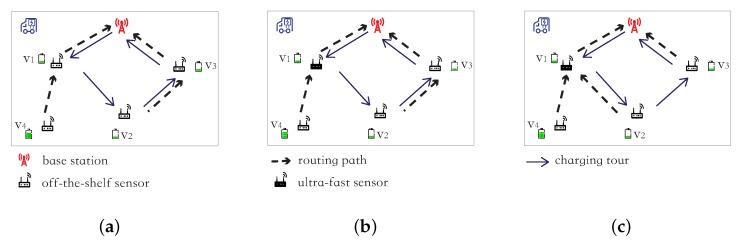
An illustration of a novel network model with deploying a few ultra-fast sensors. (**a**) a sensor network consists of only off-the-shelf sensors. (**b**) deploy one ultra-fast sensor. (**c**) allocate a new routing.

Although excellent studies have been conducted for shorten sensor dead durations, some sensors still expire their energy for some durations, as the charging time of a sensor is non-trivial (e.g., 30–80 min). For example, in Figure 1a, assume that there are four off-the-shelf sensors $v_{1}$, $v_{2}$, $v_{3}$ and $v_{4}$ in a sensor network and three of them, i.e., $v_{1}$, $v_{2}$, $v_{3}$, will run out of their energy soon. Assume that the residual lifetime $l_{i}$ of each lifetime-critical sensor $v_{i}$ is 40 min, i.e., $l_{1}=l_{2}=l_{3}=40$ min, and it takes an hour to fully charge each of them and their charging sequence is $v_{1}\to v_{2}\to v_{3}$. For the sake of convenience, we ignore the traveling time of the charging vehicle, as it is usually much shorter than the sensor charging time [27]. It can be seen that the dead durations of sensors $v_{1}$, $v_{2}$, $v_{3}$ are 0, 60−40=20 min, and 2×60−40=80 min, respectively.

On the other hand, other researchers [12,27,28,29,30] assumed that every sensor is powered with an ultra-fast charging battery, and it only takes a trivial time to fully charge such a battery, e.g., within 1 min [27,28], we here abbreviate an ultra-fast charging battery powered sensor by an ultra-fast sensor. Therefore, they usually ignored the sensor charging time when scheduling charging vehicles.

It is obvious that the adoption of ultra-fast sensors can significantly shorten sensor dead durations or even avoid their energy expirations. For example, in Figure 1a, if every sensor is powered with an ultra-fast charging battery, the energy expirations of sensors $v_{1}$, $v_{2}$, $v_{3}$ can be avoided, as the residual lifetime of each of these sensors is 40 min and it takes a very short time to charge such a sensor, e.g., 1 min.

It can be seen that, although the adoption of off-the-shelf sensors is cheap, but the dead durations of sensors may be long due to the long charging time of the sensors. Contrarily, the adoption of many ultra-fast sensors can significantly shorten sensor dead durations, but the adoption will incur a high purchasing cost. For example, the price of an ultra-fast charging battery is usually about 30–40 dollars, which is about ten times the cost of an off-the-shelf battery, e.g., a Lithium battery with the same capacity costs only 2–3 dollars [27,31]. Therefore, it is unrealistic to adopt many ultra-fast sensors in a wireless sensor network.

In this paper, we propose a novel heterogeneous sensor network model, in which there are only a few ultra-fast sensors and many low-cost off-the-shelf sensors in a sensor network. The ultra-fast sensors are deployed at some strategic locations near to the base station, as the sensors close to the base station need to relay a large amount of data from other remote sensors.

We here illustrate a heterogeneous sensor network. In Figure 1b, we only deploy one ultra-fast sensor $v_{1}$, instead of three. We now consider the charging order $v_{1}\to v_{2}\to v_{3}$ again in Figure 1b, where the residual lifetimes of the three lifetime-critical sensors $v_{1}$, $v_{2}$, $v_{3}$ are 40 min, and it takes 1 min to charge the ultra-fast sensor $v_{1}$ and an hour to fully charge either off-the-shelf sensor $v_{2}$ or $v_{3}$. Then, the dead durations of $v_{1}$, $v_{2}$, $v_{3}$ are reduced to 0, 0, and 60+1−40= 21 min, which are shorter than the sensor dead durations with only off-the-shelf sensors in Figure 1a, i.e., 0, 20 min, and 80 min.

We, however, observe that we can further reduce sensor dead durations by jointly considering charging scheduling and routing allocation in the heterogeneous sensor network. We can enable ultra-fast sensors to relay more data than off-the-shelf sensors, since the former can be quickly charged. For example, sensor $v_{2}$ can forward its data to the ultra-fast sensor $v_{1}$, rather than $v_{3}$ (see Figure 1c).

Then, the residual lifetime $l_{1}$ of $v_{1}$ is shortened from 40 min to, e.g., 20 min, while the residual lifetime $l_{3}$ of $v_{3}$ is prolonged from 40 min to, e.g., 80 min. In addition, the residual lifetime $l_{2}$ of sensor $v_{2}$ remains 40 min, as its routing load does not change. If we still charge the three lifetime-critical sensors in the order of $v_{1}\to v_{2}\to v_{3}$, none of them runs out of their energy before their energy replenishments, as their start charging time points are 0, 1st, and 61th min, respectively, while their residual lifetimes are 20, 40, and 80 min, respectively.

In this paper, we propose a novel heterogeneous network model, where a sensor network consists of only a few ultra-fast sensors and many low-cost off-the-shelf sensors. Then, the cost of deploying such a sensor network is low, due to the limited number of deployed ultra-fast sensors. On the other hand, we can significantly shorten sensor dead durations by not only scheduling the charging vehicle but also allocating routing among sensors smartly.

The main contributions in this paper are highlighted as follows:

- Unlike existing studies that either assumed that all sensors are powered with low-cost off-the-shelf batteries, or assumed that every sensor is equipped with a high-cost ultra-fast charging battery, in this paper, we propose a novel heterogeneous sensor network model, where a sensor network consists of a few ultra-fast sensors and many low-cost off-the-shelf sensors. The deployment cost of such a network then is very low.
- Under this novel network model, we study a fundamental problem of joint charging scheduling and routing allocation, such that the longest sensor dead duration is minimized. We also propose an efficient algorithm for this problem.
- We finally evaluate the performance of the proposed algorithm through extensive simulation experiments. The experimental results show that the proposed algorithm is very promising, and the longest sensor dead duration by it is only 10% of those by existing algorithms.

The rest of this paper is organized as follows: Section 2 defines the network model, charging model and the problem, Section 3 presents the algorithm for the problem, Section 4 analyzes the proposed algorithm, Section 5 evaluates the performance of the proposed algorithm through extensive simulation experiments, Section 6 introduces the related work. Finally, Section 7 concludes this paper.

## 2. Preliminaries

In this section, we first introduce a novel heterogeneous network model and then present the charging model. We finally define the problem.

### 2.1. A Novel Heterogeneous Network Model

We consider a wireless sensor network $G_{s}=\left(V_{s}\cup \left\{b\right\},E_{s}\right)$, which is deployed in a two-dimensional area, where $V_{s}$ is a set of $n_{s}$ sensors in the network, and *b* is a base station for collecting data from all sensors.

We assume that there are two types of sensors in the network. The first type of sensors are powered by off-the-shelf batteries, e.g., Lithium battery, and it usually takes a while to fully charge such a sensor, e.g., 30–80 min [26]. The other type of sensors are powered by ultra-fast charging batteries, and it takes only a very short time to fully charge such one, e.g., 1 min [27]. Assume that there are $n_{N}$ off-the-shelf sensors $v_{1},v_{2},\ldots ,v_{n_{N}}$ and $n_{F}$ ultra-fast sensors $u_{1},u_{2},\ldots ,u_{n_{F}}$. Let $V_{N}=\left\{v_{1},v_{2},\ldots ,v_{n_{N}}\right\}$ and $V_{F}=\left\{u_{1},u_{2},\ldots ,u_{n_{F}}\right\}$. Then, $V_{s}=V_{N}\cup V_{F}$. Notice that the number $n_{F}$ of ultra-fast sensors usually is very small, e.g., $n_{F}=5$, as the cost of each ultra-fast charging battery is not cheap. Denote by $c_{N}$ and $c_{F}$ the energy capacities of a sensor in $V_{N}$ and $V_{F}$, respectively.

We now consider the placement of sensors. We assume that the off-the-shelf sensors are randomly deployed. On the other hand, since the communication range of a sensor is limited, sensors close to the base station have to relay sensing data from other remote sensors. Therefore, the former sensors consume more their energy on relaying data than the latter ones. We thus assume that the ultra-fast sensors are deployed at some strategic locations near to the base station, as they can be quickly charged. For example, the ultra-fast sensor can be co-located with the most energy-consuming off-the-shelf sensors.

We assume that there is an edge $\left(v_{i},v_{j}\right)$ in $E_{s}$ for any two nodes $v_{i}$ and $v_{j}$ in $V_{s}\cup \left\{b\right\}$ if they are within the transmission range of each other. Denote by $N(v_{i})$ the set of neighbor nodes of each node $v_{i}$ in $G_{s}$, i.e, $N\left(v_{i}\right)=\left\{v_{j}|v_{j}\in V_{s}\cup \left\{b\right\},\left(v_{i},v_{j}\right)\in E_{s}\right\}$.

Assume that each sensor $v_{i}$ generates data at a rate of $r_{i}$, and the generated data will be relayed to the base station via a given routing path, e.g., the delay-aware routing [32,33]. Each sensor can monitor its residual energy level and estimate its energy consumption rate. Denote by $e_{i}$ ($e_{i}\geq 0$) the amount of residual energy of sensor $v_{i}$ at time *t*. On the other hand, following [34], the energy consumption rate of sensor $v_{i}$ at time *t* is $\rho_{i}=\mu^{s}\cdot r_{i}+\sum_{v_{j}\in N\left(v_{i}\right)}\mu_{ij}^{t}\cdot f_{ij}+\mu^{r}\cdot \sum_{v_{j}\in N\left(v_{i}\right)}f_{ji}$, where $\mu^{s}$, $\mu_{ij}^{t}$, $\mu^{r}$ are the energy consumptions for data sensing, data transmission and data reception per unit data, respectively, $f_{ij}$ and $f_{ji}$ are the data transmission rates from sensors $v_{i}$ to $v_{j}$, and from $v_{j}$ to $v_{i}$, at time *t*, respectively, $\mu_{ij}^{t}=\beta_{1}+\beta_{2}w_{i,j}^{\alpha }$, $\beta_{1}$ is the distance-independence constant term, $\beta_{2}$ is a coefficient of the distance-independence term, $w_{i,j}$ is the distance between sensor $v_{i}$ and sensor $v_{j}$, and α is the path-loss index. Then, the residual lifetime $l_{i}$ of sensor $v_{i}$ at time *t* is $l_{i}=\frac{e_{i}}{\rho_{i}}$.

### 2.2. Charging Model

As the energy stored in every sensor battery is limited, it will run out of its energy due to data sensing, data transmission and data reception. To provide controllable and perpetual energy to sensors, we employ a charging vehicle equipped with a recharging device to replenish sensor energy. Denote by $\eta_{N}$ and $\eta_{F}$ the charging rates of the vehicle for charging an off-the-shelf sensor and an ultra-fast sensor, respectively. We assume that the vehicle can move at a speed of *s* m/s.

The energy consumptions of different sensors vary significantly, i.e., some sensors may have little energy left while the others consume only a small fraction of their energy. Then, it is unnecessary for the vehicle to charge all sensors in each round, and sensors should be charged in an on-demand manner. To this end, each sensor $v_{i}$ sends a charging request to the base station once its residual lifetime $l_{i}$ falls below a given lifetime threshold $l_{c}$ at some time $t_{0}$, e.g., $l_{c}=2$ h. After receiving the charging request, the base station starts a new charging round by dispatching the vehicle to charge lifetime-critical sensors.

Let *V* be the set of to-be-charged sensors in the current charging round. There are two types of sensors in set *V*, the lifetime-critical off-the-shelf sensors in $V_{c}$ and the ultra-fast sensors in $V_{F}$, i.e., $V=V_{c}\cup V_{F}$, where an off-the-shelf sensor $v_{i}$ is contained in $V_{c}$ if its residual lifetime $l_{i}$ at time $t_{0}$ is no longer than $\lambda l_{c}$, i.e., $V_{c}=\left\{v_{i}|v_{i}\in V_{N},l_{i}\leq \lambda l_{c}\right\}$, λ is a given constant with λ≥1, and $l_{c}$ is the lifetime-critical threshold. Notice that every ultra-fast sensor in $V_{F}$ is contained in the set of to-be-charged sensors *V*, as they are deployed at strategic locations where data traffics are extremely heavy. Moreover, it only takes a very short time to fully charge an ultra-fast sensor, and the number of ultra-fast sensors $n_{F}$ usually is small, e.g., $n_{F}=5$.

**Figure 2:**
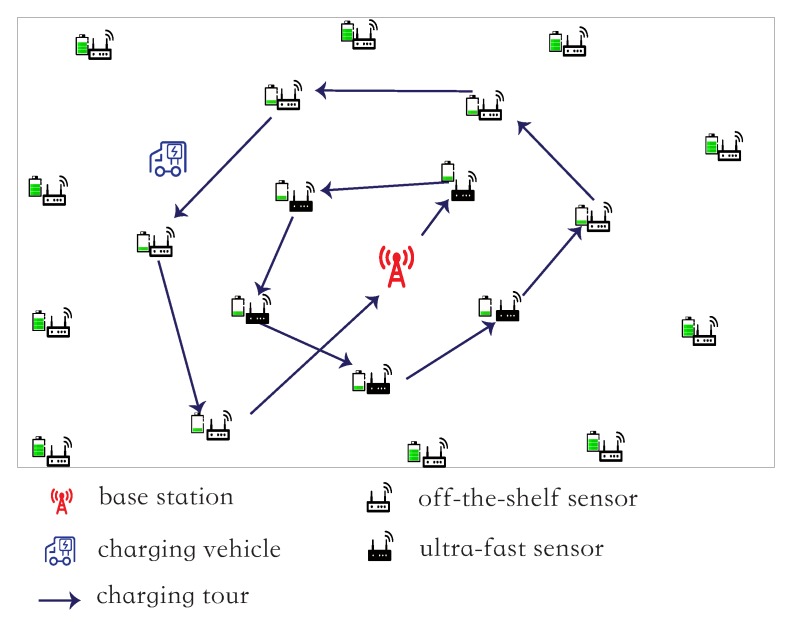
An example of a charging tour of the vehicle.

The base station will find a closed charging tour *C* of the charging vehicle for the sensors in *V* and allocate routing for the network $G_{s}$. Let $C=b\to v_{1}\to v_{2}\cdots \to v_{n_{v}}\to b$, where $n_{v}=\left|V\right|$. Then, the charging vehicle is dispatched to fully charge the sensors in *V* along tour *C* one by one (see Figure 2). Once all sensors in *V* is fully charged, the vehicle will return to the base station and recharge itself for the next charging round. Denote by *T* the duration that the vehicle spends for charging sensors along tour *C*, i.e, $T=\frac{\left(w_{b,1}+\sum_{i=1}^{n_{v}-1}w_{i,i+1}+w_{n_{v},b}\right)}{s}+\sum_{v_{j}\in V_{c}}\frac{c_{N}-e_{j}}{\eta_{N}}+\sum_{v_{k}\in V_{F}}\frac{c_{F}-e_{k}}{\eta_{F}}$, which consists of the time spent on vehicle traveling, charging the lifetime-critical off-the-shelf sensors in $V_{c}$, and charging the ultra-fast sensors in $V_{F}$.

### 2.3. Problem Definition

Many applications of sensor networks are sensitive to the data collection delay, and they usually require a continuous data collection, such as the sensor networks for video monitoring, radiation detection and forest fire detection [1,35,36]. For example, in a sensor network for radiation detection, once the energy depletion of a sensor lasts for a few hours, a radiation release cannot be detected in real time and the radiation may quickly spread to the degree of out of control and thus lead to a disaster. Therefore, we must shorten the dead durations of sensors as much as possible, due to their energy depletions. The objective of this paper is thus to find a charging tour *C* of the vehicle for charging sensors in *V* and allocate routing for each link $\left(v_{i},v_{j}\right)$ in network $G_{s}$, such that the longest dead duration of sensors in *V* is minimized. Let $t_{i}$ be the start charging time of sensor $v_{i}$ in *V* when the vehicle starts to charge sensor $v_{i}$ in tour *C*; the dead duration of sensor $v_{i}$ then is $d_{i}=t_{i}-l_{i}$ if $t_{i}>l_{i}$, where $l_{i}$ is the residual lifetime of sensor $v_{i}$ at time $t_{0}$; otherwise, ($t_{i}\leq l_{i}$), $d_{i}=0$.

Given a heterogeneous sensor network $G_{s}$, a charging vehicle, and a set *V* of to-be-charged sensors at some time $t_{0}$, the dead duration minimization problem is to find a charging tour *C* of the vehicle for charging sensors in *V*, and allocate the routing $f_{ij}$ for each link $\left(v_{i},v_{j}\right)$ in period $\left[t_{0},t_{0}+T\right]$, such that the longest dead duration of sensors in *V* is minimized, i.e.,

(1) $$\min_{f_{ij},C}\;\max_{v_{i}\in V}\left\{d_{i}\right\},$$

subject to the following constraints, i.e.,

(2) $$\begin{array}{c} \sum_{v_{j}\in N\left(v_{i}\right)}f_{ij}=r_{i}+\sum_{v_{j}\in N\left(v_{i}\right)}f_{ji},\;\forall v_{i}\in V_{s}, \end{array}$$

(3) $$\begin{array}{c} \sum_{v_{i}\in V_{s}}r_{i}=\sum_{v_{j}\in N\left(b\right)}f_{jb}, \end{array}$$

(4) $$\begin{array}{c} \rho_{i}=\mu^{s}\cdot r_{i}+\sum_{v_{j}\in N\left(v_{i}\right)}\left(\beta_{1}+\beta_{2}w_{i,j}^{\alpha }\right)\cdot f_{ij}+\mu^{r}\cdot \sum_{v_{j}\in N\left(v_{i}\right)}f_{ji},\;\forall v_{i}\in V_{s}, \end{array}$$

(5) $$\begin{array}{c} l_{i}=\frac{e_{i}}{\rho_{i}},\;e_{i}\geq 0,\forall v_{i}\in V_{s}, \end{array}$$

(6) $$\begin{array}{c} d_{i}=\max\left\{t_{i}-l_{i},0\right\},\;\forall v_{i}\in V, \end{array}$$

(7) $$\begin{array}{c} t_{i}=\left\{\begin{array}{c} t_{i-1}+\frac{c_{F}-e_{i-1}}{\eta_{F}}+\frac{w_{i-1,i}}{s},\;i\geq 2,\forall v_{i-1}\in V_{F},\forall v_{i}\in V,\\ t_{i-1}+\frac{c_{N}-e_{i-1}}{\eta_{N}}+\frac{w_{i-1,i}}{s},\;i\geq 2,\forall v_{i-1}\in V_{c},\forall v_{i}\in V. \end{array}\right. \end{array}$$

Constraint (2) shows the flow reservation of each sensor $v_{i}$. Constraint (3) indicates that the generated data from all sensors in $V_{s}$ must be sent back to the base station. Constraints (4) and (5) calculate the energy consumption rate and the residual lifetime of each sensor $v_{i}$ at time $t_{0}$, respectively. Constraints (6) and (7) define the dead duration $d_{i}$ and the start charging time $t_{i}$ of sensor $v_{i}$ in the tour, and $t_{1}=t_{0}+\frac{w_{b,1}}{s}$.

## 3. Algorithm for the Dead Duration Minimization Problem

In this section, we devise a joint charging scheduling and routing allocation algorithm for the dead duration minimization problem. We first present the framework of the algorithm, which invokes two algorithms for two subproblems of the original problem. We then detail the two algorithms.

### 3.1. Algorithm Framework

Recall that the dead duration minimization problem is to allocate the routing $f_{ij}$ for each link $\left(v_{i},v_{j}\right)$ in period $\left[t_{0},t_{0}+T\right]$, and find a charging tour *C* for the charging vehicle to replenish sensors in *V*, such that the longest dead duration of sensors is minimized. It can be seen that the allocation of routing $f_{ij}$ and the scheduling of charging tour *C* are tightly coupled. On one hand, the residual lifetime of each sensor is highly related to the routing $f_{ij}$, where a sensor consumes its energy quickly if it relays a large amount of data from other sensors. To minimize the longest sensor dead duration, the vehicle should charge the sensors with short residual lifetimes first. On the other hand, once the charging tour *C* is delivered, the start charging time that each sensor will be charged by the vehicle can be derived, by following Constraint (7). The routing $f_{ij}$ should be carefully allocated, such that the dead duration of each sensor before its start charging time is minimized. In this paper, we tackle the challenging dead duration minimization problem by considering its two subproblems. In the first subproblem, assume that the routing $f_{ij}$ is given, and the subproblem is to find a charging tour *C*, such that the longest dead duration of sensors in *V* is minimized, and this subproblem is referred to as the charging scheduling subproblem. In the second subproblem, we assume the charging tour *C* is given, and the subproblem is to allocate the routing $f_{ij}$ for each link $\left(v_{i},v_{j}\right)$, so that we can deliver the minimum longest sensor dead duration, and this problem is referred to as *the routing allocation subproblem*. We will propose two algorithms for the two subproblems in two later subsections, respectively. Having the two algorithms, we devise a joint charging scheduling and routing allocation algorithm as follows.

The algorithm iteratively finds the routing and charging tour. Denote by $f_{ij}^{K-1}$ and $f_{ij}^{K}$ the routings allocated before and after the *K*th iteration, respectively. Similarly, denote by $C^{K-1}$ and $C^{K}$ the charging tours delivered before and after the *K*th iteration, respectively, where K=1,2,…. Initially, we obtain $f_{ij}^{0}$ by assuming that each sensor uploads its data along its minimum energy path to the base station. Within each iteration *K*, the algorithm consists of two steps. In the first step, with the given routing $f_{ij}^{K-1}$, it finds a charging tour $C^{K}$, by utilizing the algorithm for the charging scheduling subproblem. In the second step, having the charging tour $C^{K}$, it obtains a routing $f_{ij}^{K}$ by invoking the algorithm for the routing allocation subproblem. If the new solution $\left(f_{ij}^{K},C^{K}\right)$ is no better than $\left(f_{ij}^{K-1},C^{K-1}\right)$, the algorithm terminates and $\left(f_{ij}^{K-1},C^{K-1}\right)$ is the final solution to the dead duration minimization problem. Otherwise, (($f_{ij}^{K},C^{K})$ is better than $(f_{ij}^{K-1},C^{K-1}$)), we continue to the next iteration, until the difference of the objective values of the two solutions is no greater than a small threshold ϵ with ϵ>0, e.g., ϵ=1 min, or the number of the performed iterations achieves a given maximum iteration number $K_{max}$, e.g., $K_{max}=100$.

The algorithm for the dead duration minimization problem is presented in Algorithm 1.
**Algorithm 1** Joint charging scheduling and routing allocation algorithm (CSRA).**Input:** a set $V_{s}$, a set *V* of to-be-charged sensors, the residual energy $e_{i}$ of each sensor $v_{i}$ in $V_{s}$ at some time $t_{0}$, a small threshold ϵ with ϵ>0.**Output:** routing $f_{ij}$, charging tour *C*.   1:Obtain a routing $f_{ij}^{0}$ by assuming that each sensor uploads its data along its minimum energy path to the base station;   2:**for**K← 1 to $K_{max}$ **do**  3: Find a charging tour $C^{K}$ with the routing $f_{ij}^{K-1}$, by invoking Algorithm 2 for the charging scheduling subproblem;   4: Obtain a routing $f_{ij}^{K}$ with the charging tour $C^{K}$, by invoking Algorithm 3 for the routing allocation subproblem;  5: Let $D^{K-1}$ and $D^{K}$ be the longest sensor dead durations of solutions $\left(f_{ij}^{K-1},C^{K-1}\right)$ and $\left(f_{ij}^{K},C^{K}\right)$, respectively;  6: **if** $D^{K}>D^{K-1}$ **then**  7:  $C\leftarrow C^{K-1},f_{ij}\leftarrow f_{ij}^{K-1}$; /*$\left(f_{ij}^{K},C^{K}\right)$ is no better than $\left(f_{ij}^{K-1},C^{K-1}\right)$ */  8:  break;  9: **else**10:  $C\leftarrow C^{K},f_{ij}\leftarrow f_{ij}^{K}$; /*$\left(f_{ij}^{K},C^{K}\right)$ is better than $\left(f_{ij}^{K-1},C^{K-1}\right)$*/11: **end if**12: **if** $D^{K-1}-D^{K}\leq \epsilon$ **then**13:  /* the difference of the longest sensor dead durations $D^{K}$ and $D^{K-1}$ of solutions $\left(f_{ij}^{K},C^{K}\right)$ and $\left(f_{ij}^{K-1},C^{K-1}\right)$ is smaller than ϵ*/ 14:  break;15: **end if**16:**end for**17:**return** charging tour *C*, and routing $f_{ij}$.

### 3.2. Algorithm for the Charging Scheduling Subproblem

Given a set $V(=V_{F}\cup V_{c})$ of to-be-charged sensors at some time $t_{0}$, and the routing $f_{ij}^{K-1}$ for each link $\left(v_{i},v_{j}\right)$ in period $\left[t_{0},t_{0}+T\right]$. The charging scheduling subproblem is to find a charging tour $C^{K}$, such that the longest dead duration of sensors in *V* is minimized, i.e.,

$$P_{1}:\;\min\;\max_{v_{i}\in V}\left\{d_{i}^{K}\right\},$$

subject to constraints (2), (3), (4), (5), (6), and (7).

Assume that the charging time of each lifetime-critical off-the-shelf sensor in $V_{c}$ is a constant δ [37], e.g., δ = 1 h, since its residual energy is very low, where $\delta \approx \frac{c_{N}}{\eta_{N}}$, $c_{N}$ is the energy capacity of the sensor, and $\eta_{N}$ is the off-the-shelf sensor charging rate. We further assume that the traveling time among two consecutive visited sensors can be considered as a small constant τ, e.g., τ=1 min, as the vehicle traveling time usually is much shorter than the charging time δ of a lifetime-critical off-the-shelf sensor in $V_{c}$, e.g., 1 min vs. 1 h [23]. Let Δ=δ+τ.

Since it takes only a very short time to fully charge ultra-fast sensors in $V_{F}$ and these sensors have heavy data relay loads, the vehicle should charge sensors in $V_{F}$ before the lifetime-critical off-the-shelf sensors in $V_{c}$. Thus, the charging tour $C^{K}$ consists of a sub-tour $C_{F}^{K}$ for charging ultra-fast sensors in $V_{F}$ and a sub-tour $C_{N}^{K}$ for replenishing lifetime-critical off-the-shelf sensors in $V_{c}$, i.e., $C^{K}=b\to C_{F}^{K}\to C_{N}^{K}\to b$. We obtain sub-tour $C_{F}^{K}$ by a brute-force search, by enumerating all charging sequences. Notice that the number $n_{F}$ of ultra-fast sensors in $V_{F}$ usually is very small, and the brute-force search then will not take a long time. On the other hand, we derive the sub-tour $C_{N}^{K}$ for lifetime-critical off-the-shelf sensors in $V_{c}$ in non-decreasing order of their residual lifetimes, i.e, $C_{N}^{K}=v_{1}\to v_{2}\to \cdots \to v_{n}$, where $l_{1}\leq l_{2}\leq \cdots \leq l_{n}$, $l_{i}$ is the residual lifetime of sensor $v_{i}$ and $n=|V_{c}|$.

The algorithm for the charging scheduling subproblem is presented in Algorithm 2. We later will showthat Algorithm 2 delivers an optimal solution to the charging scheduling subproblem (see Section 4.1).
**Algorithm 2** Algorithm for the charging scheduling subproblem.**Input:** a set *V* of to-be-charged sensors, routing $f_{ij}^{K-1}$, the residual energy $e_{i}$ of each sensor $v_{i}$. **Output:** charging tour $C^{K}$. 1:Calculate the residual lifetime $l_{q}^{K}$ of each sensor $v_{q}$ in *V*; 2:Find a charging sub-tour $C_{F}^{K}$ of ultra-fast sensors in $V_{F}$ by a brute-force search, such that the longest dead duration of sensors in $V_{F}$ is minimized; 3:Sort sensors in $V_{c}$ by their residual lifetimes in non-decreasing order, i.e., $l_{1}^{K}\leq l_{2}^{K}\leq \cdots \leq l_{n}^{K}$, where $l_{j}^{K}$ is the residual lifetime of sensor $v_{j}$ in $V_{c}$, and $n=|V_{c}|$;4:Find a charging sub-tour $C_{N}^{K}$ of sensors in $V_{c}$ by charging them in the order of $v_{1}$, $v_{2}$, …, $v_{n}$, i.e., $C_{N}^{K}=v_{1}\to v_{2}\to \cdots \to v_{n}$; 5:Let $C^{K}=b\to C_{F}^{K}\to C_{N}^{K}\to b$; 6:**return** charging tour $C^{K}$.

### 3.3. Algorithm for the Routing Allocation Subproblem

Given a sensor network $G_{s}$, a set *V* of to-be-charged sensors, and the charging tour $C^{K}$ of sensors in *V*, the routing allocation subproblem is to find the routing $f_{ij}^{K}$ for each link $\left(v_{i},v_{j}\right)$ in period $\left[t_{0},t_{0}+T\right]$, such that the longest dead duration of sensors in *V* is minimized, i.e.,

(8) $$P_{2}:\;\min_{f_{ij}^{K}}\;\left\{D^{K}\right\},$$

subject to constraints (2), (3), (4), (7), and constraint

(9) $$D^{K}=\max_{v_{i}\in V}\left\{t_{i}^{K}-\frac{e_{i}}{\rho_{i}^{K}}\right\},$$

where $D^{K}$ is the longest dead duration of sensors in *V*, $t_{i}^{K}$, $e_{i}$ and $\rho_{i}^{K}$ are the start charging time, residual energy and energy consumption rate of sensor $v_{i}$, respectively. Notice that only flow rates $f_{ij}^{K}$s are variables.

To obtain the optimal flow rates, we rewrite constraint (9) as:

(10) $$\begin{array}{c} t_{i}^{K}-\frac{e_{i}}{\rho_{i}^{K}}\leq D^{K},\;\forall v_{i}\in V, \end{array}$$

which is equivalent to $\rho_{i}^{K}\cdot t_{i}^{K}-e_{i}\leq D^{K}\rho_{i}^{K},\forall v_{i}\in V$. Then, subproblem $P_{2}$ is equivalent to

(11) $$P_{3}:\;\min_{f_{ij}}\;D^{K},$$

subject to constraints (2), (3), (4), (7), and

(12) $$\begin{array}{c} \rho_{i}^{K}\cdot t_{i}^{K}-e_{i}\leq D^{K}\rho_{i}^{K},\;\forall v_{i}\in V. \end{array}$$

We note that the objective function in problem $P_{3}$ is a linear function, and constraints (2), (3), (4), and (7) are also linear functions. However, constraint (12) is not a linear function, where $t_{i}^{K}$, and $e_{i}$ are constants, while $\rho_{i}^{K}$ and $D^{K}$ are variables.

To solve problem $P_{3}$, we reduce it to a series of linear programming (LP) problems. Denote by $D_{opt}^{K}$ the minimum longest dead duration of sensors in *V*. The basic idea behind is to guess the optimal value $D_{opt}^{K}$. Given a guess $D_{g}^{K}$ of $D_{opt}^{K}$, we consider the following LP:

$$P_{4}:1,$$

subject to constraints (2), (3), (4), (7), and

(13) $$\begin{array}{c} \rho_{i}^{K}\cdot t_{i}^{K}-e_{i}\leq D_{g}^{K}\rho_{i}^{K},\;\forall v_{i}\in V. \end{array}$$

Notice that constraint (13) is a linear function, as $D_{g}^{K}$ is a given constant.

When $D_{g}^{K}<D_{opt}^{K}$, we will show that there are no feasible solutions to LP $P_{4}$ (see Section 4.2). Otherwise ($D_{g}^{K}\geq D_{opt}^{K}$), we will prove that there is a feasible solution to LP $P_{4}$. Then, we can find the minimum longest sensor dead duration $D_{opt}^{K}$ through a binary search in interval [0,T].

The algorithm for the routing allocation subproblem is given in Algorithm 3.
**Algorithm 3** Algorithm for the routing allocation subproblem.**Input:** a set $V_{s}$ of sensors, a set *V* of to-be-charged sensors, the residual energy $e_{i}$ of each sensor $v_{i}$ in $V_{s}$ at some time $t_{0}$, the charging tour $C^{K}$. **Output:** routing $f_{ij}^{K}$.   1:Calculate the start charging time $t_{i}$ of each sensor $v_{i}$ in *V* by Constraint (7);  2:Let $D_{l}=0$ and $D_{u}=T$; /* $D_{l}$ and $D_{u}$ are the lower and upper bounds on the optimal value $D_{opt}^{K}$, respectively */   3: **while** $D_{l}<D_{u}$ **do**  4: Let $D_{g}^{K}=\left\lfloor \frac{D_{l}+D_{u}}{2}\right\rfloor$; /* a guess of $D_{opt}^{K}$ */   5: **if** there are no feasible solutions $f_{ij}^{K}$ to LP $P_{4}$ **then**  6:  /* the guess $D_{g}^{K}$ is smaller than $D_{opt}^{K}$, i.e., $D_{g}^{K}<D_{opt}^{K}$*/  7:  Let $D_{l}=D_{g}^{K}+1$;  8: **else**  9:  /* the guess $D_{g}^{K}$ is no less than $D_{opt}^{K}$, i.e., $D_{g}^{K}\geq D_{opt}^{K}$ */ 10:  Let $D_{u}=D_{g}^{K}$; 11: **end if**12:**end while**13:Let $D_{opt}^{K}=D_{u}\left(=D_{l}\right)$; 14:Find a feasible solution $f_{ij}^{K}$ to LP $P_{4}$ with $D_{opt}^{K}$; 15:**return** routing $f_{ij}^{K}$.

## 4. Algorithm Analysis

In this section, we analyze the proposed algorithms for the charging scheduling subproblem and routing allocation subproblem in Section 4.1 and Section 4.2, respectively.

### 4.1. Algorithm Analysis for the Charging Scheduling Subproblem

Recall that the objective of the charging scheduling subproblem is to find a charging tour $C^{K}$ for sensors in *V*, such that the longest dead duration of sensors in *V* is minimized, given routing $f_{ij}^{K-1}$, where tour $C^{K}$ consists of a sub-tour $C_{F}^{K}$ for charging ultra-fast sensors in $V_{F}$ and a sub-tour $C_{N}^{K}$ for replenishing lifetime-critical off-the-shelf sensors in $V_{c}$, sub-tour $C_{F}^{K}$ is obtained by a brute-force search, sub-tour $C_{N}^{K}$ is delivered by charging sensors in $V_{c}$ in non-decreasing order of their residual lifetimes, i.e., $C_{N}^{K}=v_{1}\to v_{2}\to \cdots \to v_{n}$, $l_{1}\leq l_{2}\leq \cdots \leq l_{n}$, and $n=|V_{c}|.$ In the following, we show that the sub-tour $C_{N}^{K}$ delivered by Algorithm 2 is an optimal solution to the charging scheduling subproblem, i.e., the longest dead duration of sensors in $V_{c}$ is minimized, if they are charged in non-decreasing order of their residual lifetimes, see Theorem 1.

**Theorem** **1.** *Given a set V of to-be-charged sensors, Algorithm 2 delivers an optimal solution to the charging scheduling subproblem.*

**Proof** **of** **Theorem** **1.** For the sake of convenience, assume that $l_{1}\leq l_{2}\leq \cdots \leq l_{n}$, where $l_{i}$ is the residual lifetime of a sensor $v_{i}\in V_{c}$ and $n=|V_{c}|$. Denote by $T_{F}$ the time that the charging vehicle spends in the sub-tour $C_{F}^{K}$. Then, the dead duration of $v_{i}$ in sub-tour $C_{N}^{K}$ is $d_{i}=\max\left\{T_{F}+\left(i-1\right)\Delta -l_{i},0\right\}$, where Δ=δ+τ, δ is the charging time of each lifetime-critical off-the-shelf sensor and τ is the traveling time among two consecutive visited sensors. Let $D(C_{N}^{K})$ be the longest sensor dead duration in sub-tour $C_{N}^{K}$, i.e., $D\left(C_{N}^{K}\right)=\max_{v_{i}\in V_{c}}\left\{d_{i}\right\}$. Denote by $d_{i}^{*}$ the dead duration of sensor $v_{i}$ in an optimal solution, and denote by $OPT^{K}$ the longest sensor dead duration in the optimal solution. Then, $OPT^{K}=\max_{1\leq i\leq n}\left\{d_{i}^{*}\right\}$.

In the following, we show that sub-tour $C_{N}^{K}$ is an optimal solution by an induction on the number of sensors *n* in $V_{c}$.

(*i*) Consider n=1, it is obvious that Algorithm 2 delivers an optimal solution.

(ii) We assume that Algorithm 2 can deliver an optimal solution $C_{j}^{*}$, when there are n=j sensors with j≥2.

(iii) Consider that there are n=j+1 sensors. Let $C_{N}^{K}=v_{1}\to v_{2}\to \cdots \to v_{j}\to v_{j+1}$ be the sub-tour delivered by Algorithm 2, where $l_{1}\leq l_{2}\leq \cdots \leq l_{j}\leq l_{j+1}$. We distinguish our discussion into two cases: case (1) sensor $v_{j+1}$ is charged at the last in the optimal solution; and case (2) sensor $v_{j+1}$ is not charged at the last in the optimal solution.

We first consider case (1) sensor $v_{j+1}$ is charged at the last in the optimal solution. We show that the longest sensor dead duration $OPT_{j+1}^{K}$ in an optimal solution is no shorter than that in the solution delivered by Algorithm 2, since

$$\begin{array}{ccc} OPT_{j+1}^{K} & = & \max_{1\leq i\leq j+1}\left\{d_{i}^{*}\right\},\;\mathrm{by}\;\mathrm{the}\;\mathrm{definition}\;\mathrm{of}\;OPT_{j+1}^{K},\\ & = & \max\{\max_{1\leq i\leq j}\left\{d_{i}^{*}\right\},d_{j+1}^{*}\},\\ & \geq & \max\{OPT_{j}^{K},d_{j+1}^{*}\},\;\mathrm{by}\;\mathrm{the}\;\mathrm{definition}\;\mathrm{of}\;OPT_{j}^{K},\\ & = & \max\{OPT_{j}^{K},d_{j+1}\},\;\mathrm{as}\;v_{j+1}\;\mathrm{is}\;\mathrm{charged}\;\mathrm{at}\;\mathrm{the}\;\mathrm{last}\;\mathrm{in}\;\mathrm{the}\;\mathrm{optimal}\;\mathrm{solution},\\ & = & \max\{\max_{1\leq i\leq j}\left\{d_{i}\right\},d_{j+1}\},\;\mathrm{by}\;\mathrm{the}\;\mathrm{assumption}\;\mathrm{that}\;\mathrm{Algorithm}\;2\\ & & \;\mathrm{delivers}\;\mathrm{an}\;\mathrm{optimal}\;\mathrm{solution}\;\mathrm{when}\;\mathrm{there}\;\mathrm{are}\;j\;\mathrm{sensors},\\ & = & D\left(C_{N}^{K}\right),\;\mathrm{by}\;\mathrm{the}\;\mathrm{definition}\;\mathrm{of}\;C_{N}^{K}. \end{array}$$

Therefore, $C_{N}^{K}$ is also an optimal solution.

We then consider case (2) sensor $v_{j+1}$ is charged at the *p*th order in the optimal solution $C_{j+1}^{*}$ with p≠j+1, while another sensor $v_{q}$ is charged at the (j+1)th order, i.e., $C_{j+1}^{*}=v_{1}^{*}\to v_{2}^{*}\to \cdots \to v_{j+1}\to \cdots \to v_{q}$. We obtain another charging sub-tour $C_{j+1}^{\prime }$ from $C_{j+1}^{*}$ by swapping only the charging orders of sensors $v_{q}$ and $v_{j+1}$, i.e., $C_{j+1}^{\prime }=v_{1}^{*}\to v_{2}^{*}\to \cdots \to v_{q}\to \cdots \to v_{j+1}$. We will prove that the longest sensor dead duration in $C_{j+1}^{\prime }$ is no longer than $OPT_{j+1}^{K}$, i.e., $D\left(C_{j+1}^{\prime }\right)\leq OPT_{j+1}^{K}$. On one hand,

(14) $$\begin{array}{ccc} D\left(C_{j+1}^{{}^{\prime }}\right) & = & \max_{1\leq i\leq j+1}\left\{d_{i}^{{}^{\prime }}\right\},\;\mathrm{by}\;\mathrm{the}\;\mathrm{definition}\;\mathrm{of}\;D\left(C_{j+1}^{{}^{\prime }}\right),\\ & = & \max\{\max_{v_{i}\in V_{c}\setminus \left\{v_{q},v_{g+1}\right\}}\left\{d_{i}^{{}^{\prime }}\right\},\max\left\{d_{q}^{{}^{\prime }},d_{j+1}^{{}^{\prime }}\right\}\},\\ & = & \max\{\max_{v_{i}\in V_{c}\setminus \left\{v_{q},v_{g+1}\right\}}\left\{d_{i}^{*}\right\},\max\left\{d_{q}^{{}^{\prime }},d_{j+1}^{{}^{\prime }}\right\}\}, \end{array}$$

since the charging order of each sensor $v_{i}$ in set $V_{c}\backslash \left\{v_{q},v_{g+1}\right\}$ in tour $C_{j+1}^{\prime }$ is the same as that in the optimal solution $C_{j+1}^{*}$. Note that $d_{q}^{\prime }=\max\left\{T_{F}+\left(p-1\right)\Delta -l_{q},0\right\}$, $d_{j+1}^{\prime }=\max\left\{T_{F}+j\Delta -l_{j+1},0\right\}$.

On the other hand, following the definition of the optimal value $OPT_{j+1}^{K}$, we know that

(15) $$\begin{array}{ccc} OPT_{j+1}^{K} & = & \max_{1\leq i\leq j+1}\left\{d_{i}^{*}\right\},\\ & = & \max\{\max_{v_{i}\in V_{c}\backslash \left\{v_{q},v_{g+1}\right\}}\left\{d_{i}^{*}\right\},\max\left\{d_{q}^{*},d_{j+1}^{*}\right\}\}, \end{array}$$

where $d_{q}^{*}=\max\left\{T_{F}+j\Delta -l_{q},0\right\}$, and $d_{j+1}^{*}=\max\left\{T_{F}+\left(p-1\right)\Delta -l_{j+1},0\right\}$.

It can be seen that

(16) $$\begin{array}{ccc} d_{q}^{\prime } & = & \max\{T_{F}+\left(p-1\right)\Delta -l_{q},0\},\\ & \leq & \max\{T_{F}+j\Delta -l_{q},0\},\;\mathrm{as}\;p\leq j+1,\\ & = & d_{q}^{*}, \end{array}$$

and

(17) $$\begin{array}{ccc} d_{j+1}^{\prime } & = & \max\{T_{F}+j\Delta -l_{j+1},0\},\\ & \leq & \max\{T_{F}+j\Delta -l_{q},0\},\mathrm{as}\;l_{j+1}\geq l_{q},\\ & = & d_{q}^{*}. \end{array}$$

Then, by combining Equations (16) and (17), we have that

(18) $$\begin{array}{c} \max\left\{d_{q}^{\prime },d_{j+1}^{\prime }\right\}\leq d_{q}^{*}\leq \max\left\{d_{q}^{*},d_{j+1}^{*}\right\}. \end{array}$$

Therefore, we have $D\left(C_{j+1}^{\prime }\right)\leq OPT_{j+1}^{\prime }$. Then, $C_{j+1}^{\prime }$ is also an optimal solution, where sensor $v_{j+1}$ is charged at the last. The rest is to reduce to case (1), omitted.

We finally analyze the time complexity of Algorithm 2. We first obtain a sub-tour $C_{F}^{K}$ for the ultra-fast sensors in $V_{F}$ with a brute-force search, which takes time $O(n_{F}\times n_{F}!)$, where $n_{F}=\left|V_{F}\right|$. We then find a sub-tour $C_{N}^{K}$ for the off-the-shelf sensors, by charging them in non-decreasing order of their residual lifetimes, which takes O(nlogn) time. Therefore, the time complexity of Algorithm 2 is $O(n_{F}\times n_{F}!+n\log n)$, where $n=|V_{c}|$. It must be mentioned that the number of ultra-fast sensors in a sensor network usually is very small, e.g., $n_{F}=5$. Therefore, the time complexity of Algorithm 2 is only O(nlogn). The theorem then follows. ☐

### 4.2. Algorithm Analysis for the Routing Allocation Subproblem 

In this subsection, we show that Algorithm 3 delivers an optimal solution to the routing allocation subproblem by Theorem 2.

**Theorem** **2.** *Algorithm 3 delivers an optimal solution to the routing allocation subproblem.*

**Proof** **of** **Theorem** **2.** In Section 3.3, we have shown that the routing allocation subproblem $P_{2}$ is equivalent to subproblem $P_{3}$. In the following, we show that (*i*) there are no feasible solutions to the Linear Programming (LP) $P_{4}$ when $D_{g}^{K}<D_{opt}^{K}$; and (ii) that there is a feasible solution $f_{ij}^{K}$ to LP $P_{4}$ if $D_{g}^{K}\geq D_{opt}^{K}$. Then, the optimal value $D_{opt}^{K}$ can be found through a binary search in interval [0,T].

We first show (*i*) there are no feasible solutions to LP $P_{4}$ if $D_{g}^{K}<D_{opt}^{K}$. Assume that there is a feasible solution $f_{ij}^{K}$ to LP $P_{4}$ when $D_{g}^{K}<D_{opt}^{K}$. Notice that $f_{ij}^{K}$ is also a feasible solution to subproblem $P_{3}$ and the longest sensor dead duration is $D_{g}^{K}$. However, this contradicts the assumption that $D_{opt}^{K}$ is the minimum longest sensor dead duration. Then, the assumption that there is a feasible solution $f_{ij}^{K}$ to LP $P_{4}$ is incorrect when $D_{g}^{K}<D_{opt}^{K}$.

On the other hand, we prove (ii) there is a feasible solution $f_{ij}^{K}$ to LP $P_{4}$ if $D_{g}^{K}\geq D_{opt}^{K}$. Let $f_{ij}^{*K}$ be an optimal solution to LP $P_{3}$, and $\rho_{i}^{*K}$ be the energy consumption rate of sensor $v_{i}$ with routing $f_{ij}^{*K}$. We have

$$\begin{array}{ccc} \rho_{i}^{*K}\cdot t_{i}^{K}-e_{i} & \leq & D_{opt}^{K}\rho_{i}^{*K},\\ & \leq & D_{g}^{K}\rho_{i}^{*K},\;\mathrm{as}\;D_{g}^{K}\geq D_{opt}^{K}, \end{array}$$

that is, $\rho_{i}^{*K}\cdot t_{i}^{K}-e_{i}\leq D_{g}^{K}\rho_{i}^{*K}$, which indicates $f_{ij}^{*K}$ is a feasible solution to LP $P_{4}$ if $D_{g}^{K}\geq D_{opt}^{K}$. The theorem then follows. ☐

## 5. Performance Evaluation

In this section, we evaluate the performance of the proposed algorithm CSRA through extensive simulations. We will also study the impact of important parameters on the algorithm performance, including the network size, the maximum data sensing rate, the off-the-shelf sensor charging rate, the vehicle traveling speed, and the number of ultra-fast sensors.

### 5.1. Simulation Environment

We consider a wireless sensor network deployed in a 500 m × 500 m two-dimensional square area, and a base station is located at the center of the square. There are 100 to 500 off-the-shelf sensors randomly deployed in the area, and a small number $n_{F}$ of ultra-fast sensors are co-located with the most energy-consuming off-the-shelf sensors, e.g., $n_{F}=5$. Both the capacities of an off-the-shelf battery and an ultra-fast charging battery are 10.8 kJ. A charging vehicle is initially located at the base station, and its moving speed is s=5 m/s. The charging rates $\eta_{N}$ and $\eta_{F}$ of the vehicle for charging an off-the-shelf sensor and an ultra-fast sensor are 5 W and 300 W, respectively. Then, the durations for charging an off-the-shelf sensor and an ultra-fast sensor to their full energy capacities are 36 min ($=\frac{10.8\;\mathrm{kJ}}{5\;W}$) and 36 s ($=\frac{10.8\;\mathrm{kJ}}{300\;W}$), respectively. The data sensing rate $r_{i}$ of each sensor $v_{i}$ is randomly selected from an interval $\left[r_{min},r_{max}\right]$, where $r_{min}$= 1 kbps and $r_{max}$ = 10 kbps. A well-known energy consumption model of sensors is adopted from [34]. The simulator was implemented in language C++ and all simulations were operated on a Dell server with an Intel(R) Core(TM) i7 CPU (2.5 GHz) and a 16 GB RAM (ChengDu, China). In our simulation experiments, the monitoring period of the sensor network is one year. To evaluate the performance of the proposed algorithm CSRA, we also consider five existing algorithms. Specifically, algorithm TSP (traveling salesman problem) finds a charging tour such that the vehicle traveling distance in the tour is minimized, while ignoring the sensor residual lifetimes [16]. Algorithm EDF (earliest deadline first) charges sensors in non-decreasing order of sensor residual lifetimes. Algorithm AA (Adaptive Algorithm) schedules the vehicle to charge some sensors before their energy depletions, such that the difference of the energy replenished to sensors and the energy consumed on vehicle traveling is maximized [25]. Algorithm NETWRAP (an NDN based real time wireless recharging protocol) chooses to-be-charged sensors by considering both the vehicle traveling time and the sensor residual lifetimes [26]. Finally, algorithm TSCA (temporal spatial real-time charging scheduling algorithm) first obtains a charging tour in non-decreasing order of sensor residual lifetimes, and then adjusts the sensor charging sequence, so that the number of dead sensors is minimized and the energy efficiency of the vehicle is maximized [18]. We will apply each of the mentioned algorithms in 20 different network topologies with the same network size and then obtain average values.

### 5.2. The Convergence of Algorithm CSRA

**Figure 3:**
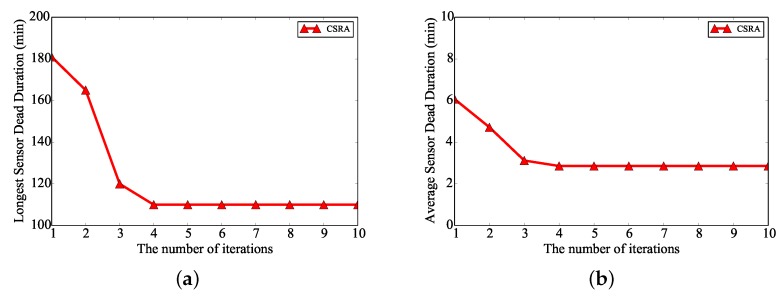
The convergence of Algorithm CSRA, when there are 200 off-the-shelf sensors. (**a**) Longest sensor dead duration. (**b**) Average sensor dead duration.

We first study the convergence of the proposed algorithm CSRA (joint Charging Scheduling and Routing Allocation algorithm), by increasing the number of iterations $K_{max}$ from 1 to 10. Figure 3 shows that the longest and average sensor dead durations by Algorithm CSRA decrease very quickly with the increase of the number of iterations $K_{max}$ and the performance of Algorithm CSRA almost does not change when $K_{max}\geq 5$.

### 5.3. Algorithm Performance 

In the following, we study the impact of the network size, the maximum data sensing rate, the off-the-shelf sensor charging rate, the vehicle traveling speed, and the number of ultra-fast sensors.

**Figure 4:**
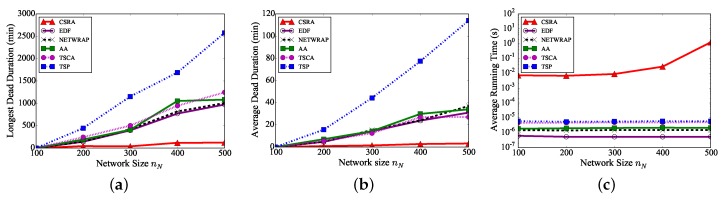
The performance of different algorithms, by varying the number of off-the-shelf sensors $n_{N}$, while $r_{max}=10$ kbps, $\eta_{N}=5$ W, and s=5 m/s. (**a**) Longest sensor dead duration. (**b**) Average sensor dead duration. (**c**) Average running times of different algorithms.

We first evaluate the performance of the proposed algorithm CSRA against existing algorithms TSP, NETWRAP, AA, TSCA, by varying the number $n_{N}$ of off-the-shelf sensors from 100 to 500, while the number $n_{F}$ of ultra-fast sensors is 5. Figure 4a shows that the longest sensor dead duration by each algorithm becomes longer with the increase of the network size, as more sensors need to be charged and their waiting times before their chargings are prolonged. Figure 4a also demonstrates that the longest sensor dead durations delivered by the proposed algorithm CSRA are much shorter than those by the existing five algorithms. For example, the longest sensor dead duration by algorithm CSRA is only about $4.3\%(\approx \frac{110}{2575})$, $11.3\%(\approx \frac{110}{973})$, $10.9\%(\approx \frac{110}{1007})$, $10.1\%(\approx \frac{110}{1083})$, and $8.8\%(\approx \frac{110}{1246})$ of those by algorithms TSP, EDF, NETWRAP, AA, TSCA, respectively, when there are 500 off-the-shelf sensors. The rationale behind is that algorithm CSRA considers both the charging scheduling and routing allocation to shorten the longest sensor dead duration in the heterogeneous sensor networks, while existing algorithms considered only the sensor residual lifetimes and vehicle traveling cost. Figure 4b further indicates that the average sensor dead durations by algorithm CSRA are also significantly shorter than those by the other five algorithms. For example, the average sensor dead duration by algorithm CSRA is about 2.8%, 10.3%, 8.6%, 9.3%, 11.8% of those by algorithms TSP, EDF, NETWRAP, AA, TSCA, respectively, when there are $\eta_{N}=500$ off-the-shelf sensors. Figure 4c shows the average running times of the six mentioned algorithms, from which it can be seen that the average running time of algorithm CSRA is longer than the five algorithms. It, however, must be mentioned that the average running time of algorithm CSRA is acceptable in practice, as it is no more than two seconds even when there are $\eta_{N}=500$ off-the-shelf sensors. In the following, we do not compare the running times of the algorithms, since the curves are similar.

**Figure 5:**
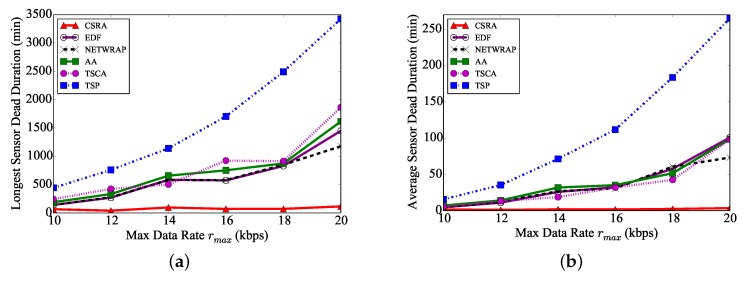
The performance of different algorithms, by varying the maximum data sensing rate $r_{max}$, while $n_{N}=200$, $\eta_{N}=5$ W, and s=5 m/s. (**a**) Longest sensor dead duration. (**b**) Average sensor dead duration.

We then investigate the performance of the algorithms, by increasing the maximum data sensing rate $r_{max}$ from 10 kbps to 20 kbps, where $n_{N}=200,s=5$ m/s, and $\eta_{N}=5$ W. Figure 5a,b show the longest and average sensor dead durations of the six algorithms increase with a larger maximum data sensing rate $r_{max}$, respectively. The rationale behind is that sensors will consume more of their energy with larger data sensing rates, and sensors then may run out of their energy faster. Figure 5 also demonstrates that the longest and average sensor dead durations by algorithm CSRA are much better than those by the existing algorithms, especially when the data sensing rate is larger. For example, the longest sensor dead duration by algorithm CSRA is only 3.4%, 8.0%, 9.8%, 9.9%, and 7.2%, 6.2% of those by algorithms TSP, EDF, NETWRAP, AA, TSCA, respectively, when $r_{max}=20$ kbps.

**Figure 6:**
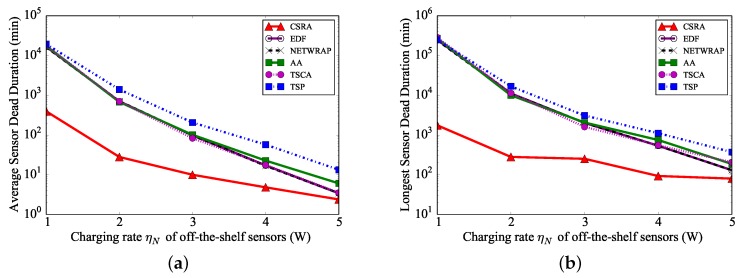
The performance of different algorithms, by varying the charging rate $\eta_{N}$ of off-the-shelf sensors, while $n_{N}=200$, $r_{max}=10$ kbps, and s=5 m/s. (**a**) Longest sensor dead duration. (**b**) Average sensor dead duration.

We also evaluate the algorithm performance, by varying the charging rate $\eta_{N}$ of an off-the-shelf sensor from 1 W to 5 W, while fixing the charging rate $\eta_{F}$ of an ultra-fast sensor at 300 W. It can be seen from Figure 6 that the longest and average sensor dead durations become shorter when the charging rate $\eta_{N}$ grows, as the waiting time of each sensor before its charging is shorter with a fast charging rate. Figure 6a further plots that the longest sensor dead duration by algorithm CSRA is the shortest, e.g., only about 5% of those by the other algorithms when the charging rate $\eta_{N}$ of off-the-shelf sensors is 1 W.

**Figure 7:**
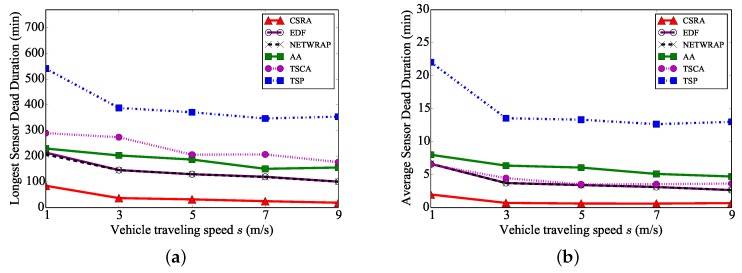
The performance of different algorithms, by varying the vehicle traveling speed *s*. (**a**) Longest sensor dead duration. (**b**) Average sensor dead duration.

We further study the impact of the vehicle traveling speed *s* on the algorithm performance, by varying the speed *s* from 1 m/s to 10 m/s. Figure 7 plots that the longest and average sensor dead durations of the proposed algorithm CSRA are still the shortest ones among the six algorithms. Figure 7 also shows that the longest dead duration by each of the six algorithms only slightly decreases with a faster vehicle traveling speed. The improvement is only slight, since the sensor charging time is much longer than the vehicle traveling time [23,26].

**Figure 8:**
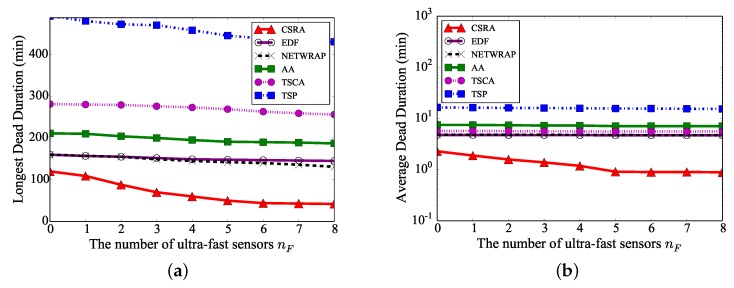
The performance of different algorithms, by increasing the number of ultra-fast sensors $n_{F}$ from 0 to 8, while there are 200 off-the-shelf sensors. (**a**) Longest sensor dead duration. (**b**) Average sensor dead duration.

We finally investigate the performance of different algorithms, by increasing the number of ultra-fast sensors $n_{F}$ from 0 to 8, while there are 200 off-the-shelf sensors. Figure 8a demonstrates that the longest sensor dead duration by Algorithm CSRA decreases with the increase of $n_{F}$. Notice that the longest sensor dead duration by Algorithm CSRA is only about 70% of those by the existing algorithms when there are no ultra-fast sensors, i.e., $n_{F}=0$, since Algorithm CSRA jointly considers charging scheduling and routing allocation by enabling other off-the-shelf sensors that have sufficient energy to relay more data for lifetime-critical sensors, while existing algorithms only considered the charging scheduling of mobile charging vehicles. Figure 8 also shows that the longest and average sensor dead durations by Algorithm CSRA only slightly decease when there are more than five ultra-fast sensors, and the longest sensor dead duration by Algorithm CSRA is about 25% of those by the existing algorithms when $n_{F}=5$.

In summary, it can be seen from the experimental results that the longest and average sensor dead durations by the proposed algorithm CSRA are much shorter than those by existing algorithms TSP, EDF, NETWRAP, AA and TSCA.

## 6. Related Work

The employment of mobile charging vehicles is a promising technique to replenish sensor energy, and the vehicle scheduling for prolonging network lifetimes has been studied in literature. Most of these studies assumed that every sensor is powered with a low-cost off-the-shelf battery [17,18,19,20,21,23,38,39,40], e.g., Lithium battery, where the price of such a battery is about only a few dollars [31]. It, however, usually takes some non-trivial time to fully charge such a battery, e.g., 30–80 min [26].

Some studies employed only one charging vehicle to charge sensors in a sensor network [17,18,19,20,23]. He et al. [17] proposed a Nearest-Job-Next with Preemption discipline to increase the average number of charged sensors per unit time and shorten their charging latencies. Liang et al. [20] proposed approximation algorithms to maximize the total utility of charging sensors in a charging tour, subject to the energy capacity of a charging vehicle. Lin et al. [18] devised a temporal-spatial charging scheduling algorithm to minimize the number of dead sensors and maximize energy efficiency. Xu et al. [23] maximized sensor lifetimes while minimizing the length of charging tour, by proposing a novel charging paradigm in which each sensor can be partially replenished. Lin et al. [19] developed a Primary and Passer-by scheduling algorithm to increase the number of charged sensors before their energy expiration times, by charging some energy-sufficient sensors that are close to energy-critical sensors, such that their chargings will not prolong the dead durations of latter sensors.

On the other hand, some other studies [21,38,39,40] employed multiple charging vehicles to maintain sensor networks perpetually. Liang et al. [40] devised an approximation algorithm to minimize the number of dispatched vehicles to charge energy-critical sensors, subject to the energy capacity of each vehicle. Jiang et al. [39] considered not only the charging sequence but also the vehicle depot positioning, such that the number of deployed charging vehicles is minimized and the ratio of the sensor charging time to traveling time is maximized. Dai et al. [38] scheduled multiple charging vehicles to replenish sensor energy with an objective of maximizing the total amount of energy charged into sensors, and further minimizing the overall charging time under the electromagnetic radiation safety constraint. Wang et al. [21] proposed a hybrid network framework that consists of solar-powered sensors and wireless-powered sensors. They studied the problem of minimizing vehicle traveling energy, such that the perpetual operations of sensors are maintained.

It can be seen that, although excellent studies have been conducted for replenishing sensor energy and shortening their dead durations, some sensors may still expire their energy, as the charging time of a sensor is non-trivial, e.g., 30–80 min [26].

We note that other researchers [12,27,28,29,30] assumed that every sensor is powered with an ultra-fast charging battery, and it only takes a trivial time to fully charge such a battery, e.g., within 1 min [27,28]. Therefore, they usually ignored the sensor charging time. For example, Zhao et al. [28] assumed that a vehicle is able to not only charge sensors but also collect sensing data. In each charging round, they first found the charging tour of lifetime-critical sensors and then allocated routing in the period that the vehicle performs sensor charging, such that the utility of collected sensing data is maximized. Zhang et al. [29] adopted a collaborative charging paradigm, in which any two charging vehicles can transfer energy with each other. They investigated the problem of employing multiple vehicles to charge sensors, such that the ratio of the energy consumed for charging sensors to the energy consumption on vehicle traveling is maximized. Xu et al. [27] studied the problem of scheduling multiple charging vehicles to charge sensors in a given monitoring period such that none of these sensors depletes its energy and the total traveling cost of the vehicles is minimized, where the energy consumption rates of different sensors vary significantly.

Different from these mentioned existing studies, in this paper, we propose a novel heterogeneous network model by deploying a few ultra-fast sensors at some strategic locations near the base station. The network cost is low, since we deploy only a few expensive ultra-fast sensors. On the other hand, we show that the network performance can be significantly improved with even such a small number of ultra-fast sensors, by enabling ultra-fast sensors to relay more data for other lifetime-critical sensors.

## 7. Conclusions

Unlike exiting studies that deployed only off-the-shelf sensors or ultra-fast sensors, in this paper, we proposed a novel heterogeneous sensor network model, in which a sensor network consists of a few ultra-fast sensors and many low-cost off-the-shelf sensors. Then, the deployment cost of the network is low as the number of ultra-fast sensors is limited. Under the novel network model, we studied a problem of finding a charging tour and allocating routing, such that the longest sensor dead duration is minimized. We further devised an efficient algorithm for the problem, by enabling ultra-fast sensors to relay more data from lifetime-critical off-the-shelf sensors. We finally evaluated the proposed algorithm through extensive simulations. In addition, experimental results showed that the longest sensor dead duration by the proposed algorithm is much shorter than those by existing algorithms, e.g., 90% shorter.

## References

[1] Cheng X., Yang N., Shi Y., Lei H. Design of radiation detection system with WSN. Proceedings of the 2011 Cross Strait Quad-Regional Radio Science and Wireless Technology Conference.

[2] IEC White Paper Internet of Things: Wireless Sensor Networks. http://www.iec.ch/whitepaper/pdf/iecWP-internetofthings-LR-en.pdf.

[3] Kim S., Pakzad S., Culler D., Demmel J., Fenves G., Glaser S., Turon M. Health monitoring of civil infrastructures using wireless sensor networks. Proceedings of the 6th international conference on Information processing in sensor networks.

[4] Werner-Allen G., Lorincz K., Welsh M., Marcillo O., Johnson J., Ruiz M., Lees J. (2006). Deploying a wireless sensor network on an active volcano. IEEE Internet Comput..

[5] Yu L., Wang N., Meng X. Real-time forest fire detection with wireless sensor networks. Proceedings of the 2005 International Conference on Wireless Communications, Networking and Mobile Computing.

[6] Keshavarzian A., Lee H., Venkatraman L. Wakeup scheduling in wireless sensor networks. Proceedings of the 7th ACM international symposium on Mobile ad hoc networking and computing.

[7] Ye W., Heidemann J., Estrin D. An energy-efficient MAC protocol for wireless sensor networks. Proceedings of the Twenty-First Annual Joint Conference of the IEEE Computer and Communications Societies.

[8] Zhang Z., Ma M., Yang Y. (2005). Energy efficient multi-hop polling in clusters of two-layered heterogeneous sensor networks. IEEE Trans. Comput..

[9] Voigt T., Ritter H., Schiller J. Utilizing solar power in wireless sensor networks. Proceedings of the 28th Annual IEEE International Conference on Local Computer Networks.

[10] Wang C., Guo S., Yang Y. Energy-efficient mobile data collection in energy-harvesting wireless sensor networks. Proceedings of the 20th IEEE International Conference on Parallel and Distributed Systems (ICPADS).

[11] Rahimi M., Shah H., Sukhatme G., Heideman J., Estrin D. Studying the feasibility of energy harvesting in a mobile sensor network. Proceedings of the 2003 IEEE International Conference on Robotics and Automation.

[12] Xu W., Liang W., Lin X., Mao G. (2016). Efficient scheduling of multiple mobile chargers for wireless sensor networks. IEEE Trans. Veh. Technol..

[13] Karalisa A., Joannopoulosb J.D., Soljacicb M. (2008). Efficient wireless non-radiative mid-range energy transfer. Ann. Phys..

[14] Kurs A., Karalis A., Moffatt R., Joannopoulos J.D., Fisher P., Soljacic M. (2007). Wireless power transfer via strongly coupled magnetic resonances. Science.

[15] Wang C., Li J., Ye F., Yang Y. Improve charging capability for wireless rechargeable sensor networks using resonant repeaters. Proceedings of the IEEE 35th International Conference on Distributed Computing Systems.

[16] Xie L., Shi Y., Hou Y.T., Sherali H.D. (2012). Making sensor networks immortal: An energy-renewal approach with wireless power transfer. IEEE/ACM Trans. Netw..

[17] He L., Kong L., Gu Y., Pan J., Zhu T. (2015). Evaluating the on-demand mobile charging in wireless sensor networks. IEEE Trans. Mob. Comput..

[18] Lin C., Zhou J., Guo C., Song H., Wu G., Obaidat M.S. (2018). TSCA: A temporal-spatial real-time charging scheduling algorithm for on-demand architecture in wireless rechargeable sensor networks. IEEE Trans. Mob. Comput..

[19] Lin C., Han D., Deng J., Wu G. (2017). P2S: A primary and passer-by scheduling algorithm for on-demand charging architecture in wireless rechargeable sensor networks. IEEE Trans. Veh. Technol..

[20] Liang W., Xu W., Shi W., Mao G., Das S.K. (2017). Approximation algorithms for charging reward maximization in rechargeable sensor networks via a mobile charger. IEEE/ACM Trans. Netw..

[21] Wang C., Li J., Ye F., Yang Y., Ye F. A hybrid framework combining solar energy harvesting and wireless charging for wireless sensor networks. Proceedings of the IEEE INFOCOM 2016—The 35th Annual IEEE International Conference on Computer Communications.

[22] Xie L., Shi Y., Hou Y.T., Lou W., Sherali H.D., Midkiff S.F. (2015). Multi-node wireless energy charging in sensor networks. IEEE/ACM Trans. Netw..

[23] Xu W., Liang W., Jia X., Xu Z. (2018). Maximizing sensor lifetime with the minimal service cost of a mobile charger in wireless sensor networks. IEEE Trans. Mob. Comput..

[24] Wang C., Li J., Ye F., Yang Y. Recharging schedules for wireless sensor networks with vehicle movement costs and capacity constraints. Proceedings of the Eleventh Annual IEEE International Conference on Sensing, Communication, and Networking.

[25] Wang C., Li J., Ye F., Yang Y. (2016). A mobile data gathering framework for wireless rechargeable sensor networks with vehicle movement costs and capacity constraints. IEEE Trans. Comput..

[26] Wang C., Li J., Ye F., Yang Y. (2014). NETWRAP: An NDN based real-timewireless recharging framework for wireless sensor networks. IEEE Trans. Mob. Comput..

[27] Xu W., Liang W., Lin X., Mao G., Ren X. Towards perpetual sensor networks via deploying multiple mobile wireless chargers. Proceedings of the 43rd International Conference on Parallel Processing.

[28] Zhao M., Li J., Yang Y. (2014). A framework of joint mobile energy replenishment and data gathering in wireless rechargeable sensor networks. IEEE Trans. Mob. Comput..

[29] Zhang S., Wu J., Lu S. Collaborative mobile charging for sensor networks. Proceedings of the IEEE 9th International Conference on Mobile Ad-Hoc and Sensor Systems.

[30] Zhao M., Li J., Yang Y. Joint mobile energy replenishment and data gathering in wireless rechargeable sensor networks. Proceedings of the 23rd International Teletraffic Congress.

[31] Lithium Battery. https://www.amazon.com/Energizer-Lithium-Batteries-Lasts-Longer/dp/B00003IEMD/ref=sr_1_1_s_it?s=hpc&ie=UTF8&qid=1534904877&sr=1-1&keywords=Energizer+AA+Lithium+Batteries+2+Pack.

[32] Boshoff J.N., Helberg A.S.J. Improving QoS for real-time multimedia traffic in ad-hoc networks with delay aware multi-path routing. Proceedings of the 2008 Wireless Telecommunications Symposium.

[33] Yao Y., Cao Q., Vasilakos A.V. (2015). EDAL: An energy-efficient, delay-aware, and lifetime-balancing data collection protocol for heterogeneous wireless sensor networks. IEEE/ACM Trans. Netw..

[34] Hou Y.T., Shi Y., Sherali H.D. (2008). Rate allocation and network lifetime problems for wireless sensor networks. IEEE/ACM Trans. Netw..

[35] Barbarán J., Diaz M., Esteve I., Rubio B. (2007). RadMote: A mobile framework for radiation monitoring in nuclear power plants. Int. J. Electron. Circuit. Syst..

[36] Gomaa R., Adly I., Sharshar K., Safwat A., Ragai H. ZigBee wireless sensor network for radiation monitoring at nuclear facilities. Proceedings of the 6th Joint IFIP Wireless and Mobile Networking Conference (WMNC).

[37] Zou T., Xu W., Liang W., Peng J., Cai Y., Wang T. (2017). Improving charging capacity for wireless sensor networks by deploying one mobile vehicle with multiple removable chargers. Ad Hoc Netw..

[38] Dai H., Ma H., Liu A.X. (2018). Radiation constrained scheduling of wireless charging tasks. IEEE/ACM Trans. Netw..

[39] Jiang G., Lam S.K., Sun Y., Tu L., Wu J. (2017). Joint charging tour planning and depot positioning for wireless sensor networks using mobile chargers. IEEE/ACM Trans. Netw..

[40] Liang W., Xu W., Ren X., Jia X., Lin X. Maintaining sensor networks perpetually via wireless recharging mobile vehicles. Proceedings of the 39th Annual IEEE Conference on Local Computer Networks.

